# Factors for Negative Result in Serum Anti-Helicobacter pylori IgG Antibody Test in Adult Subjects With Nodular Gastritis: A Single-center Study

**DOI:** 10.7759/cureus.15651

**Published:** 2021-06-14

**Authors:** Kyoichi Adachi, Kanako Kishi, Utae Sakamoto, Tomoko Mishiro, Eiko Okimoto, Norihisa Ishimura, Shunji Ishihara

**Affiliations:** 1 Health Center, Shimane Environment and Health Public Corporation, Matsue, JPN; 2 Second Department of Internal Medicine, Shimane University Faculty of Medicine, Izumo, JPN; 3 Gastroenterology, Shimane University Hospital, Izumo, JPN

**Keywords:** nodular gastritis, helicobacter pylori, serologic test, gi endoscopy, endoscopic finding

## Abstract

Aim: Nodular gastritis has been demonstrated to be strongly associated with *Helicobacter pylori* infection. The present retrospective study was performed to elucidate factors related to a negative serum antibody test result in adults with nodular gastritis.

Materials and methods: We investigated 116 *H. pylori*-positive subjects endoscopically diagnosed with nodular gastritis and subjected to a serum anti-*H. pylori* immunoglobulin G (IgG) antibody test. The degree of gastric mucosal atrophy and the presence of spotty redness in the gastric body and fornix were carefully determined by observations of endoscopic images.

Results: Of the 116 investigated subjects, 108 were positive and 8 negative in serum anti-*H. pylori* IgG antibody test results. The degree of gastric mucosal atrophy was mild in seven among eight seronegative cases. The levels of pepsinogen II in serum in patients with negative antibody test findings were significantly lower as compared to those found positive, while the pepsinogen I/II ratio tended to be higher in subjects shown negative by the test. Only 1 of 69 with spotty redness was negative in serum anti-*H. pylori* IgG antibody testing, while 7 of 47 without spotty redness were negative. Multiple logistic regression analysis of subjects with a negative test result revealed that the absence of spotty redness shown by endoscopy was a significant risk factor.

Conclusion: The absence of spotty redness, which may reflect the degree of gastric body inflammation, is a significant factor indicating increased risk for a negative serum anti-*H. pylori* IgG antibody test result in subjects with nodular gastritis.

## Introduction

Nodular gastritis is endoscopically characterized by an unusual small granulated pattern, initially described as a “gooseflesh phenomenon” [1]. The histological features of nodular gastritis are hyperplasia of lymphoid follicles with germinal centers in the proper lamina of the stomach [1,2]. Several investigators have demonstrated that the condition is strongly associated with *Helicobacter pylori* infection in both children and adults, and nearly, all individuals with nodular gastritis shown by endoscopy are considered to have an *H. pylori* infection [1,3-6].

An *H. pylori* infection is known to cause several types of gastrointestinal diseases, including gastritis, peptic ulcers, and gastric cancer [7-9], thus various diagnostic methods have been developed to precisely diagnose that infection [10-12]. Among the available methods, a serologic test for *H. pylori* is easily performed using obtained serum samples for both epidemiologic studies involving large numbers of subjects as well as in clinical practice for individual patients. The recently introduced anti-*H. pylori* immunoglobulin G (IgG) antibody detection kit SphereLight H. pylori antibody J® (Fujifilm Wako Pure Chemical Industries, Ltd., Osaka, Japan) was developed using antigens from *H. pylori* strains derived from Japanese patients. The sensitivity of this kit was demonstrated to be higher than that of another antibody detection kit popularly used in Japan [13-16]. However, we have found that some patients without a past history of eradication therapy for *H. pylori* show a negative result in the SphereLight *H. pylori* antibody J test, even though they have endoscopic evidence of nodular gastritis. Therefore, we performed the present retrospective study to elucidate factors related to a negative result in serum anti-*H. pylori* IgG antibody test in adults with nodular gastritis.

## Materials and methods

**Figure 1:**
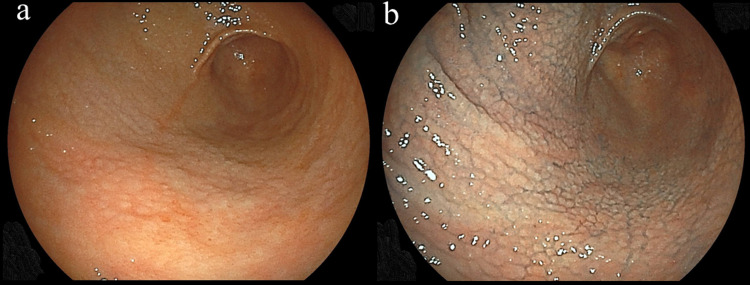
Endoscopic images of antral nodular gastritis. (a) White light image and (b) image after Indigo carmine spraying. The presence of nodular gastritis could be easily identified after Indigo carmine spraying.

The present subjects were individuals who visited the Health Center of Shimane Environment and Health Public Corporation for a detailed medical checkup examination between April 2014 and March 2018, since the newly developed *H. pylori* IgG antibody kit, SphereLight *H. pylori* antibody J®, was started to use for detection of serum *H. pylori* antibody from April 2014 in our institute. The majority were socially active and productive and considered to be socioeconomically middle class. During the study period, 17,727 subjects (11,664 males, 6,063 females; mean age 52.8 years) underwent an upper GI endoscopic examination, of whom 147 (61 males, 86 females) were endoscopically diagnosed with nodular gastritis (Figure 1). Of these 147 subjects, 125 were investigated by serum anti-*H. pylori* IgG antibody test in our institute. Of these 125 cases, 108 were diagnosed as positive for *H. pylori* infection and 17 showed negative anti-*H. pylori* IgG antibody test. We recommended 17 cases, whose anti-*H. pylori* IgG antibody test were negative, to perform other diagnostic methods, such as urea breath test or stool antigen test. Eight cases were diagnosed as positive of *H. pylori* infection by urea breath test (n=2), histology (n=2), or following anti-*H. pylori* IgG antibody test (n=4). The other two cases were not diagnosed as positive of *H. pylori* infection even after performing other diagnostic methods. We could not determine the status of *H. pylori* infection in seven cases, since they did not undergo the following medical checkup in our institute. Therefore, 116 *H. pylori*-positive cases with nodular gastritis were analyzed in this study. None had a history of eradication therapy for *H. pylori* infection, which was carefully confirmed by a trained public health nurse. In addition, none had received an anti-secretory medication, such as proton pump inhibitors and H2 receptor antagonists, nor had severely abnormal findings in renal and liver function testing.

Detection of the serum anti-*H. pylori* IgG antibody was performed using a SphereLight H. pylori antibody J® kit. The antibody titer was automatically measured using a chemiluminescent enzyme immunoassay method, with a concentration of ≥4.0 U/ml defined as positive, according to the manufacturer’s instruction sheet. The sensitivity and specificity of this cut-off value were reported to be 97.5% and 98.6%, respectively [16]. Serum levels of pepsinogen I and II were voluntarily investigated at medical checkups in 20 cases among 108 study subjects. Therefore, we analyzed these levels and the pepsinogen I/II ratio in those cases. Serum levels of pepsinogen I and II were examined by chemiluminescent enzyme immunoassay method with SphereLight pepsinogen I and II kits.

All upper endoscopic examinations were performed by licensed experienced endoscopists using an EG-530NW, EG-530NP, or EG-L580NW endoscope (Fujifilm, Tokyo, Japan). A diagnosis of nodular gastritis was confirmed when the characteristic small granulated pattern was observed in the stomach, with indigo carmine spraying performed to detect its presence in all suspected cases (Figure 1).

The degree of gastric mucosal atrophy was evaluated using the classification of Kimura and Takemoto, in which gastric mucosal atrophy is classified into six groups (C1, C2, C3, O1, O2, O3) [17]. Three expert endoscopists simultaneously reviewed endoscopic images from all subjects, and the diagnosis of nodular gastritis and degree of gastric mucosal atrophy were determined by consensus.

Statistical analyses were performed using chi-square, Fisher’s exact test, Mann-Whitney U tests, and multiple logistic regression analysis. All calculations were done using the Stat View 5.0 software program (Abacus Concepts Inc., Berkeley, CA, USA) for Macintosh, with P<0.05 considered to indicate statistical significance.

This study was performed in accordance with the Declaration of Helsinki, and the protocol was approved by the ethics committee of the Shimane Environment and Health Public Corporation (IRB no. 2017-3). Written informed consent indicating that clinical data would be used for a clinical study without the release of individual information that was obtained from all subjects before performing the medical checkup examinations.

## Results

Of the 116 *H. pylori*-infected study subjects with nodular gastritis shown by endoscopy, 108 were positive and 8 were negative in a serum anti-*H. pylori* IgG antibody test. The characteristics of subjects with positive and negative results in the antibody test are shown in Table 1.

**Table 1:** Comparison between positive and negative serum anti-H. pylori antibody test results in subjects with nodular gastritis shown by endoscopy. Values are expressed as the mean ± SD or number of subjects. Gastric mucosal atrophy was evaluated using the classification of Kimura and Takemoto (C1–C2: mild, C3–O1: moderate, O2–O3: severe gastric mucosal atrophy). ^†^Pepsinogen I, II, and the ratio of I/II were examined in 16 cases shown positive and four cases shown negative in serum anti-H. pylori antibody test results. ^‡^Spotty multiple tiny reddish spots in fundic gland region. Typical spotty redness was defined as tiny reddish lesions <1 mm in diameter occurring in abundance on the cardiac side of the fundic gland region.

	Serum anti-H. pylori antibody test		P-value
	Positive	Negative	
Gender (male/female)	42/66	4/4	0.711
Age (years)	45.0±8.0	41.5±2.7	0.293
Gastric mucosal atrophy			0.437
Mild	76	7	
Moderate to severe	32	1	
Pepsinogen I (ng/ml)^†^	80.0±24.3	64.3±14.6	0.219
Pepsinogen II (ng/ml)^ †^	19.4±7.7	10.1±2.9	0.023
Pepsinogen I/II ratio^†^	4.6±1.7	6.5±0.6	0.052
Spotty redness^‡^			0.00
Presence	68	1	
Absence	40	7	

The degree of gastric mucosal atrophy was mild in seven among eight seronegative cases. Only 1 subject with a higher graded (moderate, severe) gastric mucosal atrophy had a negative serum anti-*H. pylori* IgG antibody test result, whereas 7 (8.4%) of 83 with mild gastric mucosal atrophy had negative test results. Additionally, the levels of pepsinogen II in serum in patients with negative antibody test findings were significantly lower as compared to those found positive, while the pepsinogen I/II ratio tended to be higher in subjects shown negative by the test. The distribution of serum titers revealed by the anti-*H. pylori* IgG antibody test in these subjects is shown in Table 2.

**Table 2:** Distribution of serum titers of anti-H. pylori antibody in subjects with endoscopic nodular gastritis. Values are expressed as the number of subjects. Numbers in parentheses indicate the percentage. ^†^Gastric mucosal atrophy was evaluated using the classification of Kimura and Takemoto (C1–C2: mild, C3–O1: moderate, O2–O3: severe gastric mucosal atrophy).

Serum antibody titer	Total	Gastric mucosal atrophy^†^		
		Mild	Moderate	Severe
<1.0	0	0	0	0
1.0–1.9	1 (0.8)	1 (1.2)	0	0
2.0–2.9	1 (0.8)	1 (1.2)	0	0
3.0–3.9	6 (5.2)	5 (6.0)	1 (3.1)	0
4.0–9.9	18 (15.5)	17 (20.5)	1 (3.1)	0
10.0–39.9	59 (50.9)	38 (45.8)	20 (62.5)	1 (100)
≥40.0	31 (26.7)	21 (25.3)	10 (31.3)	0

**Figure 2:**
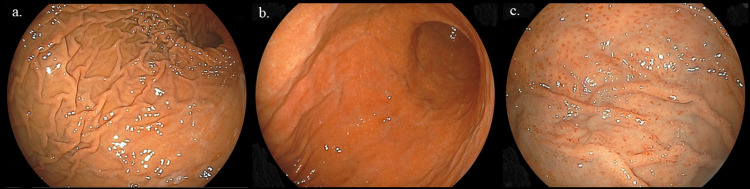
Endoscopic images of the cases with and without H. pylori infection. (a) Normal gastric body without diffuse redness in cases without *H. pylori* infection, (b) diffuse redness of gastric body in cases with *H. pylori* infection, and (c) spotty redness of fornix in the case with *H. pylori* infection. The presence or absence of diffuse redness could not be clearly determined endoscopically, whereas the presence of spotty redness could be easily identified.

**Figure 3:**
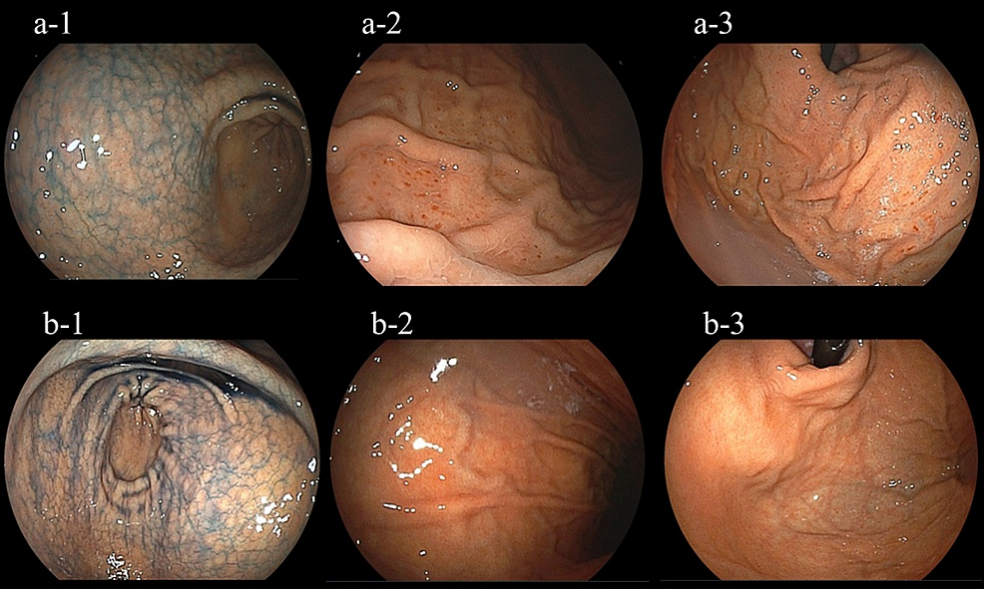
Endoscopic images of representative subjects with nodular gastritis. Case 1: Representative subject, a 48-year-old male. Endoscopy revealed antral nodular gastritis based on indigo carmine spraying findings (a-1), and the presence of spotty redness in the body (a-2) and fornix (a-3). Case 2: Representative subject, a 39-year-old female. Endoscopy revealed antral nodular gastritis based on indigo carmine spraying findings (b-1), and the absence of spotty redness in the body (b-2) and fornix (b-3). Serum anti-*H. pylori* antibody test in case 1 was positive and that in case 2 was negative.

Subjects with an *H. pylori* infection have been reported to have several characteristic endoscopic findings, including diffuse redness, spotty redness, mucosal swelling, enlarged folds, and sticky mucous, as well as gastric mucosal atrophy and nodular changes [5,18-20]. However, endoscopic findings of mucosal swelling, enlarged folds, and sticky mucous were rarely observed in the present subjects with nodular gastritis, and the presence or absence of diffuse redness could not be clearly determined, whereas we noted spotty redness in the body and fornix of the stomach shown by endoscopy (Figures 2 and 3). Such an endoscopic finding of spotty redness was observed in 69 (59.5%) of our 116 subjects, of whom only 1 (1.4%) was negative in the serum anti-*H. pylori* IgG antibody test. In contrast, 7 (14.9%) of the 47 without spotty redness were negative in the antibody test. Thus, there was a significant difference in the rate of positive findings between subjects with and without spotty redness (Table 1). In multiple logistic regression analysis findings of subjects negative for the serum anti-*H. pylori* antibody test, the absence of spotty redness in the stomach shown by endoscopy was a significant risk factor for a negative antibody test result (Table 3).

**Table 3:** Multiple logistic regression analysis of subjects found negative in serum anti-H. pylori antibody testing. ^†^Gastric mucosal atrophy was evaluated using the classification of Kimura and Takemoto (C1–C2: mild, C3–O1: moderate, O2–O3: severe gastric mucosal atrophy). Odds ratios were calculated using mild gastric mucosal atrophy in comparison with moderate and severe.

	Odds ratio	95%CI	P-value
Gender (male)	1.700	0.357-8.092	0.505
Age (increment of 1 year)	0.924	0.817-1.044	0.204
Mild gastric mucosal atrophy^†^	0.497	0.034-7.269	0.609
Spotty redness (absence)	16.475	1.366-198.755	0.027

## Discussion

An *H. pylori* infection can lead to a variety of gastrointestinal diseases, such as gastritis, peptic ulcers, and gastric cancer [7-9], thus eradication therapy is widely recommended to prevent their occurrence [21-23]. As a result, it is very important to accurately diagnose *H. pylori* infection in clinical situations, with several different invasive and non-invasive methods available [10-12]. Among those, serologic tests are widely used for both epidemiologic studies and in clinical practice, as they are easily performed in a relatively short period of time with obtained serum. Furthermore, the sensitivity and specificity for the diagnosis of *H. pylori* infection have been reported to range from 80% to 90% [10]. The diagnostic accuracy of serological tests for *H. pylori* in Japanese subjects has been repeatedly demonstrated to increase when using kits derived from antigens of *H. pylori* strains obtained from Japanese patients [24-26]. The SphereLight *H. pylori* antibody J test used in the present study is also produced using antigens from *H. pylori* strains derived from Japanese patients and has been shown to have a high efficacy for diagnosis of infection [13-15]. Here, we investigated factors causing a negative result in that test in adult cases with endoscopic nodular gastritis.

Nodular gastritis is characterized in endoscopy findings by an unusual small granulated pattern, termed the gooseflesh phenomenon, which is a common endoscopic manifestation of *H. pylori* infection in children [2-4,6]. Miyamoto et al. observed nodular gastritis in 0.19% of 97,262 individuals aged 16 years and older who underwent an upper gastrointestinal endoscopy examination [1]. In the present study, of 17,727 individuals who underwent an upper gastrointestinal endoscopy as part of their medical checkup for screening, 147 (0.83%) were diagnosed with nodular gastritis. Thus, the prevalence of this condition may be higher than previously reported and the majority of individuals affected by nodular gastritis may not have severe upper GI symptoms requiring a visit to a medical clinic. Although the prevalence of nodular gastritis shown by endoscopy is lower in adults as compared to children, the condition is recognized as a characteristic *H. pylori*-positive finding in all ages [1,5]. In addition, adults with this condition have been shown to have an increased risk of gastric cancer occurrence [27].

Based on the above information, we consider that an accurate diagnosis of *H. pylori* infection is very important, especially for adults with nodular gastritis. However, 8 (7.0%) of the present 116 subjects with that condition had a negative result in a serum anti-*H. pylori* IgG antibody testing, although these cases were diagnosed as positive of *H. pylori* infection by additional diagnostic methods, such as urea breath test, histology, or following anti-*H. pylori* IgG antibody test. Thus, different diagnostic methods should be performed when the cases with nodular gastritis show negative results of serum anti-*H. pylori* IgG antibody testing. We noted that a mild degree of gastric mucosal atrophy tended to have a correlation with a negative serum anti-*H. pylori* IgG antibody test result in the present subjects with nodular gastritis, although this tendency was not observed by the multiple logistic regression analysis. It was also suggested by the results of our analysis of the pepsinogen I/II ratio, which reflects the degree of gastric mucosal atrophy [28,29]. Previous studies have noted that gastric mucosal atrophy progression is correlated with aging [17,28-29], while *H. pylori* infection generally occurs during childhood [30]. Therefore, the duration of *H. pylori* infection might have an effect on the serum titer of anti-*H. pylori* antibody test, though the age was not significantly different between subjects with and without a positive result in the present cohort. Future study is needed to investigate the adequate cutoff value in subjects with different gastric mucosal conditions, such as several degrees of mucosal atrophy and inflammation.

Another interesting observation in the present study was that the presence of spotty redness shown by endoscopy had a significant effect on serum anti-*H. pylori* IgG antibody test results. Spotty redness is an important endoscopic finding that reflects *H. pylori* infection-induced inflammation of gastric mucosa [5,18-20]. A previous prospective multicenter study reported spotty redness in the gastric body in 70.3% of their subjects with an *H. pylori* infection [5]. In the present investigation, that prevalence was 59.5% in 116 *H. pylori*-positive subjects with nodular gastritis, a value lower in comparison with previous reports. An absence of spotty redness was well correlated with a negative finding in the serum anti-*H. pylori* antibody test. Furthermore, the level of pepsinogen II has been demonstrated to reflect the degree of gastric mucosal inflammation [29], while that in our subjects shown negative in antibody testing was significantly lower as compared to those with a positive result. Therefore, a low grade of gastric body mucosal inflammation is considered to be one of the important reasons that anti-*H. pylori* antibody test shows negative results in cases with nodular gastritis. Further study is needed to clarify the significance of spotty redness in subjects with *H. pylori* infection.

The present study has several limitations. We utilized a single serum anti-*H. pylori* IgG antibody test to evaluate the status of *H. pylori* infection and did not employ other diagnostic methods, as this was a retrospective analysis of individuals who visited a medical center for a detailed medical checkup. In addition, a majority of the present subjects were socially active and productive and considered to be socioeconomically middle class, thus young and elderly individuals were relatively few. Additional large-scale investigations employing other types of anti-*H. pylori* IgG antibody tests are needed to clarify the present observations, especially in *H. pylori*-infected children, since the majority of those have been demonstrated to have nodular gastritis in endoscopic findings.

## Conclusions

We analyzed the serum anti-*H. pylori* IgG antibody test result and several upper GI endoscopic findings in 116 subjects with nodular gastritis closely related to *H. pylori* infection. Eight of the investigated subjects showed negative results of serum anti-*H. pylori* IgG antibody test and endoscopic characteristics in those cases were mild degrees of gastric mucosal atrophy and absence of spotty redness in the gastric body and fornix, which may reflect the degree of gastric inflammation. Multiple logistic regression analysis revealed that the absence of spotty redness was a significant risk factor for a negative serum anti-*H. pylori* IgG antibody test result. It should be recognized that serum anti-*H. pylori* IgG antibody test is sometimes negative in cases with nodular gastritis when the endoscopic finding of spotty redness is not observed.
